# Eicosapentaenoic Acid (EPA) Alleviates Oxidative Stress and Inflammatory Responses in Canine Ileal Epithelial Cells

**DOI:** 10.3390/vetsci13080765

**Published:** 2026-07-31

**Authors:** Xiaoyue Yuan, Xizhu Zhu, Chuyang Zhu, Yunhan Wang, Chenhui Li, Minghua Yu, Fanfan Meng, Ping Hu, Haoyu Liu, Qu Chen, Demin Cai

**Affiliations:** 1 Laboratory of Animal Physiology and Molecular Nutrition, Jiangsu Key Laboratory of Animal Genetics, Breeding and Molecular Design, College of Animal Science and Technology, Yangzhou University, Yangzhou 225009, China; 2 Yangzhou Weike Bio-Engineering Co., Ltd., Sinopharm Group, Yangzhou 225127, China; 3 Guangling College, Yangzhou University, Yangzhou 225009, China

**Keywords:** eicosapentaenoic acid, canine ileal epithelial cells, oxidative stress, cellular senescence, intestinal barrier

## Abstract

Fueled by the rapid growth of the global companion animal industry, the development of nutritional additives for improving canine intestinal health has emerged as a prominent research area in pet nutrition. Excessive reactive oxygen species accumulation triggers severe oxidative stress, inducing intestinal inflammation, abnormal epithelial senescence, and tight junction destruction to damage intestinal mucosal integrity. Omega-3 polyunsaturated fatty acids (ω-3 PUFAs) have proven antioxidative and anti-inflammatory potency, yet divergent functions of eicosapentaenoic acid (EPA) and docosahexaenoic acid (DHA) on canine ileal epithelial cells (cIECs) remain unclear. Here, an H_2_O_2_-stimulated oxidative injury model was built to compare their protective capacity. Under all equivalent gradient concentrations ranging from 2.5 to 10 μM in this experiment, Equal-dose DHA failed to alleviate cell damage, while EPA effectively rescued impaired cIEC viability. Biochemical tests showed EPA lowered malondialdehyde and boosted key antioxidant enzyme activities. Transcriptomics confirmed EPA suppressed overactivated TNF-α/IFN-γ inflammatory and senescence pathways. Western blot verified EPA downregulated p-Chk1, p53, p57, and p73 and restored telomere-protective and tight junction proteins. In summary, EPA protects cIECs via antioxidation, anti-inflammation and anti-senescence, supporting its development as a promising functional feed additive for canine intestinal care.

## Simple Summary

This study explored the protective function and underlying molecular mechanism of eicosapentaenoic acid (EPA) against hydrogen peroxide-induced oxidative damage in canine ileal epithelial cells (cIECs), with docosahexaenoic acid (DHA) set as the parallel control. Cell viability screening tests demonstrated that at all gradient concentrations from 2.5 to 10 μM used in this experiment, DHA failed to relieve oxidative injury, while EPA exhibited prominent cytoprotective effects. Mechanistic assays confirmed that EPA alleviated intracellular redox imbalance via improving antioxidant enzyme activities, inhibited excessive inflammatory signaling, suppressed oxidative stress-initiated cellular senescence by stabilizing the telomere shelterin complex, and restored tight junction protein expression to repair impaired intestinal epithelial barrier. In conclusion, EPA is a promising natural bioactive nutrient for canine intestinal protection, laying theoretical support for the development of targeted functional feed additives for companion dogs.

## 1. Introduction

Statistics released by the American Veterinary Medical Association show that 45.5% of American households owned pet dogs and 32.1% owned pet cats in 2024 [1]. The 2026 China Pet Industry White Paper (Consumption Report) records that the number of pet dogs in China’s urban areas reached 53.43 million in 2026, an increase of 850,000 or 1.6% compared with 2024 [2]. With the growing companion value of pet dogs, scientifically balanced nutrition and refined feeding management have become the mainstream model of modern canine husbandry [3]. Among all canine health issues, intestinal health has drawn widespread attention as it profoundly regulates systemic physiological homeostasis. The intestine acts not only as the central organ responsible for nutrient digestion and absorption in dogs, but also as the largest mucosal immune barrier against the invasion of exogenous pathogens [4,5]. However, dogs are frequently exposed to various stressors during feeding, which easily trigger oxidative stress caused by excessive accumulation of reactive oxygen species (ROS), further inducing inflammatory responses, cellular injury and destruction of the intestinal epithelial barrier [6]. Oxidative stress arises when the endogenous antioxidant system fails to eliminate excess ROS under harmful stimuli, disrupting cellular redox homeostasis, initiating lipid peroxidation and impairing cell membrane integrity [7]. Therefore, developing safe and natural feed additives to alleviate oxidative damage, suppress inflammation and restore intestinal barrier function is of great importance for maintaining intestinal health in dogs.

Omega-3 polyunsaturated fatty acids (ω-3 PUFAs) can remodel membrane properties by integrating into cell membrane phospholipids and modulating multiple signaling pathways [8]. In the metabolic cascade of ω-3 PUFAs, these fatty acids competitively inhibit the activities of cyclooxygenase and lipoxygenase, reducing the production of pro-inflammatory eicosanoids such as prostaglandin E_2_ and leukotriene B_4_ [9]. Meanwhile, they markedly suppress the activation of the NF-κB signaling pathway and activate receptors including PPAR-α, PPAR-γ and GPR120, blocking the inflammatory signaling cascade and forming a multi-target anti-inflammatory network [10,11]. Eicosapentaenoic acid (EPA) and docosahexaenoic acid (DHA) are two extensively investigated ω-3 PUFAs in canine nutrition [12]. EPA has been verified to exert definite auxiliary intervention effects on inflammatory diseases of dogs such as atopic dermatitis, osteoarthritis and chronic kidney disease [13]. Although the general health benefits of DHA and EPA have been partially elucidated, their effects and underlying molecular mechanisms in alleviating intestinal oxidative stress damage in dogs remain unclear.

On this basis, the present study established an in vitro intestinal oxidative damage model by treating canine ileal epithelial cells with H_2_O_2_, compared the intestinal protective effects of EPA and DHA, and elucidated their multi-target mechanisms in relieving oxidative injury, inhibiting cellular senescence and restoring intestinal barrier integrity. This research aims to provide a theoretical basis for developing functional feed additives targeting the regulation of canine intestinal health.

## 2. Materials and Methods

### 2.1. Cell Culture and Treatment

Ileum tissues from ethically approved newborn Beagle fetuses (No.2025DA018) with pathogen-negative dams. Segments 3–8 cm from the ileocecal valve were minced, washed, and cultured as tissue explants; 40 s differential trypsinization removed fibroblasts for ≥90% epithelial purity. Immortalized *hTERT*-CIECs were passaged to P50 without senescence, with steady proliferation and >90% post-thaw viability. Multi-index microbial detection every 10 or 20 passages confirmed no contamination across P1–P50. All experiments were performed using cells at passages 2 to 4. Canine ileal epithelial cells (cIECs) were cultured in high-glucose (25 mmol/L) DMEM (Cytiva, Shanghai, China) supplemented with 5% fetal bovine serum (Hyclone, Logan, UT, USA), 10 ng/mL epidermal growth factor (Tongli Haiyuan, Beijing, China), 5 μg/mL insulin (Vicent, Saint-Jean-Baptiste, QC, Canada), 20 mM HEPES (Yuanye, Shanghai, China), and 1% penicillin–streptomycin (Solarbio, Beijing, China) at 37 °C in a 5% CO_2_ atmosphere. Cells were divided into six treatment groups: (1) blank control group (DMEM vehicle), (2) H_2_O_2_ group (400 μM H_2_O_2_; Guangdong Hengjian Pharmaceutical Co., Ltd., Guangdong, China), (3) EPA single treatment group (5 μM EPA; Sikejie, Shandong, China) for 30 h, (4) DHA single treatment group (5 μM DHA; Sikejie, Shandong, China) for 30 h, (5) EPA + H_2_O_2_ group: cells were pretreated with EPA for 24 h followed by 6 h of H_2_O_2_ stimulation, and (6) DHA + H_2_O_2_ group: cells were pretreated with DHA for 24 h followed by 6 h of H_2_O_2_ stimulation. Three replicate wells were set for each group for subsequent functional assays.

### 2.2. Calcein Staining and Live Cell Quantification with ImageJ

IECs were pretreated with 5 μM EPA or 5 μM DHA for 24 h [11], then challenged with 400 μM or 600 μM H_2_O_2_ for 6 h. The 400 μM concentration was chosen as the standard oxidative insult, as it reduces viability without causing massive necrosis, aligning with established epithelial injury models [14]. The higher 600 μM dose, which induced severe cell death, was used solely to compare the efficacy of EPA and DHA under more intense stress. The medium was removed, and cells were washed twice with assay buffer. Cells were then incubated with calcein AM (1 μmol/L) at 37 °C for 30 min. After incubation, cells were washed twice with phosphate-buffered saline (PBS), sealed with mounting medium, observed and photographed under a fluorescence microscope, and images were saved. Live cell numbers were quantified using ImageJ software.

### 2.3. Cell Viability Assay

Cells were seeded in 96-well plates at 5 × 10^3^ cells per well and pretreated with EPA/DHA (2.5, 5, 10 μM) for 24 h; the combined treatment group was then exposed to 400 μM H_2_O_2_ for 6 h. Then, 10 μL of CCK-8 solution was added to each well, and the plate was incubated in the dark for 3 h. Absorbance at 450 nm was measured using a multimode microplate reader (Tecan, Männedorf, Switzerland).

### 2.4. Measurement of Antioxidant Indices

After the completion of cell treatment, the culture medium was removed, and cells were washed with PBS and lysed on ice to extract total protein. Cell lysates were centrifuged at 12,000 rpm at 4 °C, and the supernatant was collected for protein quantification using a BCA protein assay kit (Phygene, Fuzhou, China). The levels of malondialdehyde (MDA), total superoxide dismutase (T-SOD), catalase (CAT), and glutathione peroxidase (GSH-Px) were determined using commercial assay kits (Nanjing Jiancheng Bioengineering Institute, Nanjing, China) in accordance with the manufacturer’s protocols. All results were normalized to the total protein concentration of each sample.

### 2.5. RNA-Sequencing and qRT-PCR

cIECs were seeded in 6-well plates at a density of 1.5 × 10^5^ cells/well and cultured for 24 h. cIECs were seeded in 10 mm culture dishes at a density of 6 × 10^5^ cells per dish and incubated for 24 h. The total duration of intervention for all cells was 30 h, and the treatment regimens of each group were as follows: the blank control group received an equal volume of medium solvent; the H_2_O_2_ group was treated with 400 μM H_2_O_2_ for 6 h; the EPA single treatment group was cultured with 5 μM EPA for 30 h; the EPA + H_2_O_2_ co-treatment group was pretreated with 5 μM EPA for 24 h, followed by stimulation with 400 μM H_2_O_2_ for 6 h. After treatment, cells were washed twice with PBS and harvested. Total RNA was extracted using TRIzol reagent (1 mL/well; Invitrogen, Waltham, MA, USA) following the manufacturer’s instructions and stored at −80 °C for subsequent use. RNA integrity and quality were evaluated using an Agilent 2100 Bioanalyzer (Agilent Technologies, Santa Clara, CA, USA). High-throughput transcriptome sequencing was performed on the MGI T7 platform by Wuhan Yingzi Gene Technology Co., Ltd. (Wuhan, China). Raw reads were filtered using fastp (v0.23.4) to remove adapters and low-quality bases. Quality control was performed with FastQC (v0.11.9). Clean reads were aligned to the Canis familiaris reference genome (CanFam3.1) using STAR (v2.7.10a). Gene expression quantification (FPKM) was conducted using RSEM (v1.3.3). The Q30 percentage exceeded 94.5%, and uniquely mapped rates ranged from 87.4% to 91.6%. Functional enrichment analyses, including Gene Ontology (GO) and Kyoto Encyclopedia of Genes and Genomes (KEGG) pathway annotation, were performed using the clusterProfilerpackage (v4.2.2). The org.Canis.familiaris.eg.dbannotation database was utilized for ID conversion. Gene Set Enrichment Analysis (GSEA) was conducted using GSEA software (v4.1.0) to evaluate the enrichment of hallmark pathways and cellular senescence gene sets. Statistical significance for enrichment was defined as FDR q < 0.05. Visualization of enrichment results (bubble plots, GSEA curves) was generated using the Bioinformatics platform (http://www.bioinformatics.com.cn/). Gene expression levels were quantified based on the fragments per kilobase of exon per million mapped fragments (FPKM) algorithm. Extracted RNA was reverse-transcribed into complementary DNA (cDNA) using a reverse transcription kit (Vazyme, Nanjing, China). Quantitative real-time PCR (qRT-PCR) was performed with corresponding kits (Vazyme, Nanjing, China). The relative mRNA expression levels were calculated via the 2^−^ΔΔCT method.

### 2.6. Gene Enrichment Analysis

Gene set enrichment analysis (GSEA 4.1.0) was used to identify significantly enriched pathways. In addition, statistically enriched analysis and classification of differentially expressed genes (DEGs) were performed using the Metascape database (http://metascape.org/, accessed on 7 November 2025) and DAVID (https://davidbioinformatics.nih.gov/, accessed on 7 November 2025). GSEA plots and KEGG enrichment bubble charts were generated using an online tool (http://www.bioinformatics.com.cn/, accessed on 14 November 2025).

### 2.7. Senescence-Associated β-Galactosidase Staining

cIECs were seeded in 6-well plates at a density of 1.5 × 10^5^ cells per well and cultured for 24 h to achieve stable cell attachment. The total treatment duration for all groups was 30 h. The blank control group was treated with an equal volume of culture medium solvent. The H_2_O_2_ model group was exposed to 400 μM H_2_O_2_ for 6 h. The EPA single treatment group was cultured with 5 μM EPA throughout the entire 30 h period. For the EPA + H_2_O_2_ co-treatment group, cells were pretreated with 5 μM EPA for 24 h followed by stimulation with 400 μM H_2_O_2_ for another 6 h. The culture medium was removed, cells were washed once with PBS, and β-galactosidase fixative was added for 15 min at room temperature. The fixative was removed, and cells were washed three times with PBS for 3 min each. Fresh SA-β-gal staining working solution was added to each well, and the plates were incubated overnight at 37 °C under atmospheric conditions without CO_2_. Finally, cellular senescence was observed and imaged under an optical microscope. For delayed observation, cells were immersed in PBS and stored at 4 °C.

### 2.8. Western Blot Analysis

Total protein was extracted from cIECs using lysis buffer supplemented with protease inhibitors (Biosharp, Hefei, China). After protein quantification via the BCA method (Phygene, Fuzhou, China), protein samples were normalized to equal concentrations and mixed with 5× protein loading buffer (Affinibody, Wuhan, China) at a volume ratio of 4:1. Samples were denatured by boiling at 100 °C for 10 min and stored at −20 °C for standby use. Sodium dodecyl sulfate-polyacrylamide gel electrophoresis (SDS-PAGE) was performed with concentrated and separating gels of appropriate concentrations. Electrophoresis was run at 80 V for sample stacking and then adjusted to 120 V until the bromophenol blue indicator reached the bottom of the gel. Polyvinylidene fluoride (PVDF) membranes (Millipore, Burlington, MA, USA) were activated with anhydrous methanol, and protein transfer was conducted at 100 V for 2 h in an ice-water bath. After transfer, membranes were blocked with 5% non-fat milk for 1 h at room temperature and washed three times with TBST (10 min per wash). Membranes were incubated with antibodies overnight at 4 °C, including TERF1 Rabbit pAb (ABclonal, A0137, Wuhan, China), TERF2 Rabbit pAb (ABclonal, A16316), POT1 Rabbit mAb (Zenbio, R25421, Durham, NC, USA), Phospho-Chk1-S345 Rabbit mAb (ABclonal, A21009), p53 Rabbit pAb (Zenbio, 345567), p57 Kip2 Rabbit mAb (Zenbio, R380868), p73 Rabbit mAb (Zenbio, R381172), ZO-1 Rabbit pAb (Proteintech, 21773-1-AP, Rosemont, IL, USA), Occludin Rabbit pAb (Proteintech, 27260-1-AP), JAM-1 Rabbit pAb (Proteintech, 16992-1-AP), and β-actin Rabbit pAb (Huaxing Bio, HX1831, Beijing, China); all primary antibodies were diluted at 1:1000 with antibody dilution buffer. Following three TBST washes, membranes were incubated with corresponding secondary antibodies for 1 h at room temperature. After thorough washing, ECL chemiluminescence reagent (Theorell, Wuhan, China) was added, and membranes were incubated in the dark for 2 min. Protein bands were visualized and captured using a chemiluminescence imaging system (Tannon, Shanghai, China). ImageJ software (v1.54f, NIH, Bethesda, MD, USA) was used to extract the gray values of bands for senescence-related proteins and tight junction proteins for subsequent statistical analysis.

### 2.9. Statistical Analysis

One-way analysis of variance (one-way ANOVA) was used to compare four experimental groups, namely the control group, single treatment group, H_2_O_2_-induced model group and combined treatment group. The analyzed indicators included viable cell count, CCK-8 cell viability, antioxidant biochemical parameters, and relative mRNA expression levels quantified by qRT-PCR. Two-way analysis of variance (two-way ANOVA) was specifically adopted for concentration gradient screening experiments (with two independent variables: fatty acid concentration gradient and H_2_O_2_ stimulation) and quantitative analysis of gray values of Western blot bands. Tukey’s multiple range post hoc test for pairwise comparisons between individual groups. Statistical significance thresholds were set at * *p* < 0.05, ** *p* < 0.01, and *** *p* < 0.001.

## 3. Results

### 3.1. EPA Ameliorated H_2_O_2_-Induced Viable Cell Reduction in cIECs

**Figure 1:**
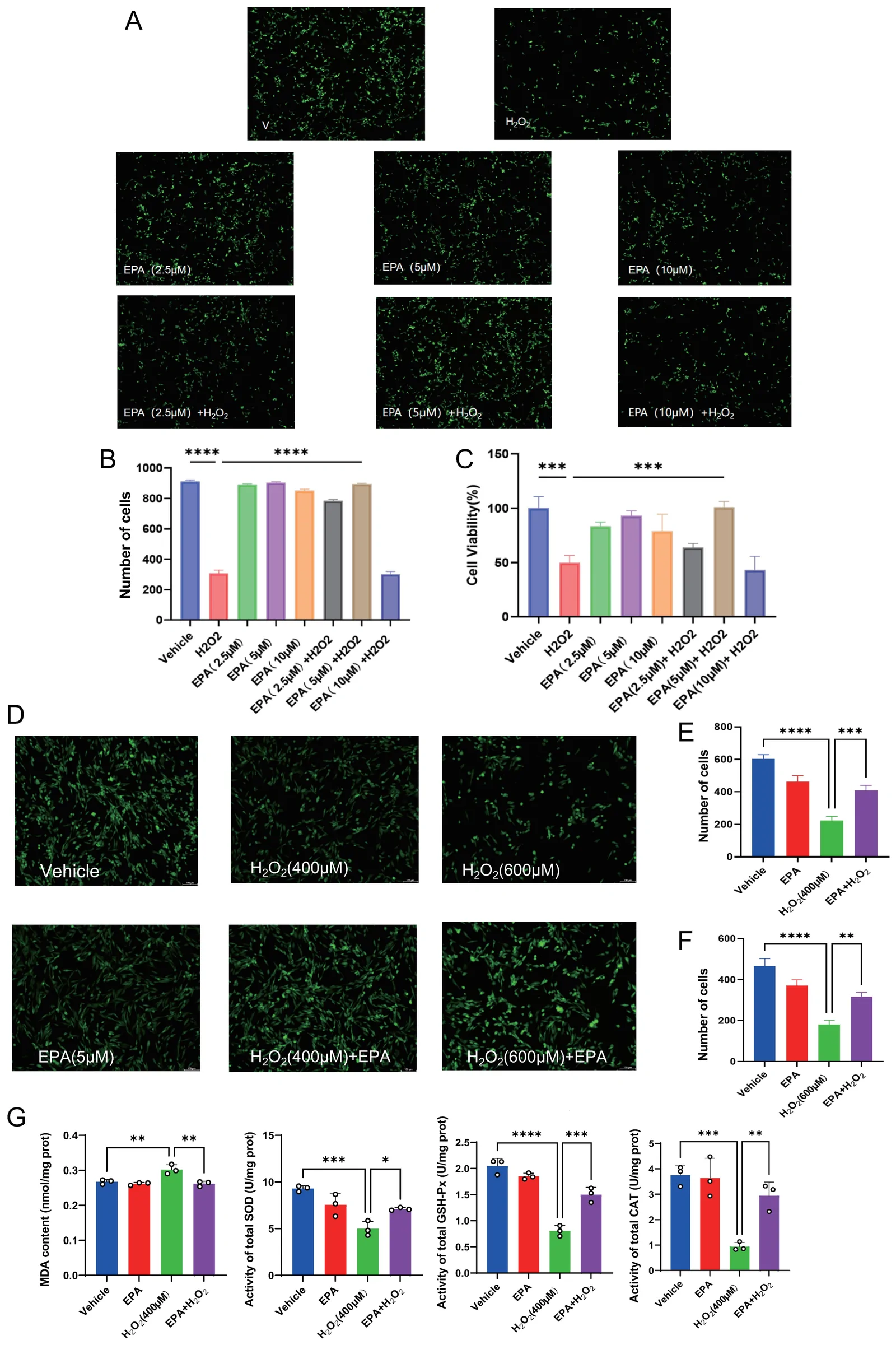
Calcein-AM fluorescence staining and quantitative statistics of viable cIECs following EPA pretreatment against H_2_O_2_-induced injury. (**A**) Representative Calcein-AM fluorescence images of cells in the Vehicle, 400 μM H_2_O_2_ alone, 2.5/5/10 μM EPA alone, and 2.5/5/10 μM EPA + 400 μM H_2_O_2_ groups. (**B**) Statistical quantification of viable cell counts across different EPA concentration gradients; (**C**) CCK-8-based cell viability detection for the EPA concentration gradient. (**D**) Representative fluorescence images of cells in the Vehicle, 400 μM H_2_O_2_, 600 μM H_2_O_2_, 5 μM EPA alone, EPA + 400 μM H_2_O_2_, and EPA + 600 μM H_2_O_2_ groups. (**E**,**F**) Quantitative viable cell counts for 5 μM EPA intervention under 400 μM and 600 μM H_2_O_2_ challenge, respectively. (**G**) Quantitative detection of MDA content and enzymatic activities of total-SOD, GSH-Px and CAT in each experimental group. Data are presented as mean ± SD (*n* = 3 per group); * *p* < 0.05, ** *p* < 0.01, *** *p* < 0.001, **** *p* < 0.0001. Scale bar = 100 μm.

Calcein-AM live cell fluorescence staining and CCK-8 assay were performed to evaluate the cytoprotective efficacy of EPA against H_2_O_2_-induced oxidative damage in cIECs. Three gradient concentrations (2.5, 5, and 10 μM) of EPA were applied for 24 h pretreatment prior to 400 μM H_2_O_2_ stimulation. As shown in Figure 1A–C, exposure to 400 μM H_2_O_2_ resulted in a dramatic reduction in both viable cell count and overall cell viability compared with the Vehicle group (*p* < 0.001). EPA pretreatment restored cell survival, and 5 μM EPA achieved the optimal cytoprotective effect. Accordingly, 5 μM was selected as the optimal working concentration of EPA for all subsequent mechanistic experiments. We further verified the protective effect of 5 μM EPA under two different oxidative stress intensities (400 μM and 600 μM H_2_O_2_). As presented in Figure 1D–F, EPA pretreatment significantly increased viable cell numbers under both concentrations of H_2_O_2_ (*p* < 0.01), confirming its stable cytoprotective activity against oxidative injury.

Subsequent biochemical detection of oxidative stress-related indicators revealed dramatically elevated MDA content and significantly reduced enzymatic activities of total-SOD, CAT and GSH-Px in the H_2_O_2_-treated group relative to the Vehicle group (Figure 1G). After 5 μM EPA pretreatment, intracellular MDA accumulation in cIECs was efficiently restricted, accompanied by prominent recovery of SOD, CAT and GSH-Px activities (*p* < 0.05). Collectively, EPA effectively restored the impaired endogenous antioxidant system in oxidatively injured cIECs.

### 3.2. DHA Failed to Ameliorate H_2_O_2_-Induced Viable Cell Reduction in cIECs

**Figure 2:**
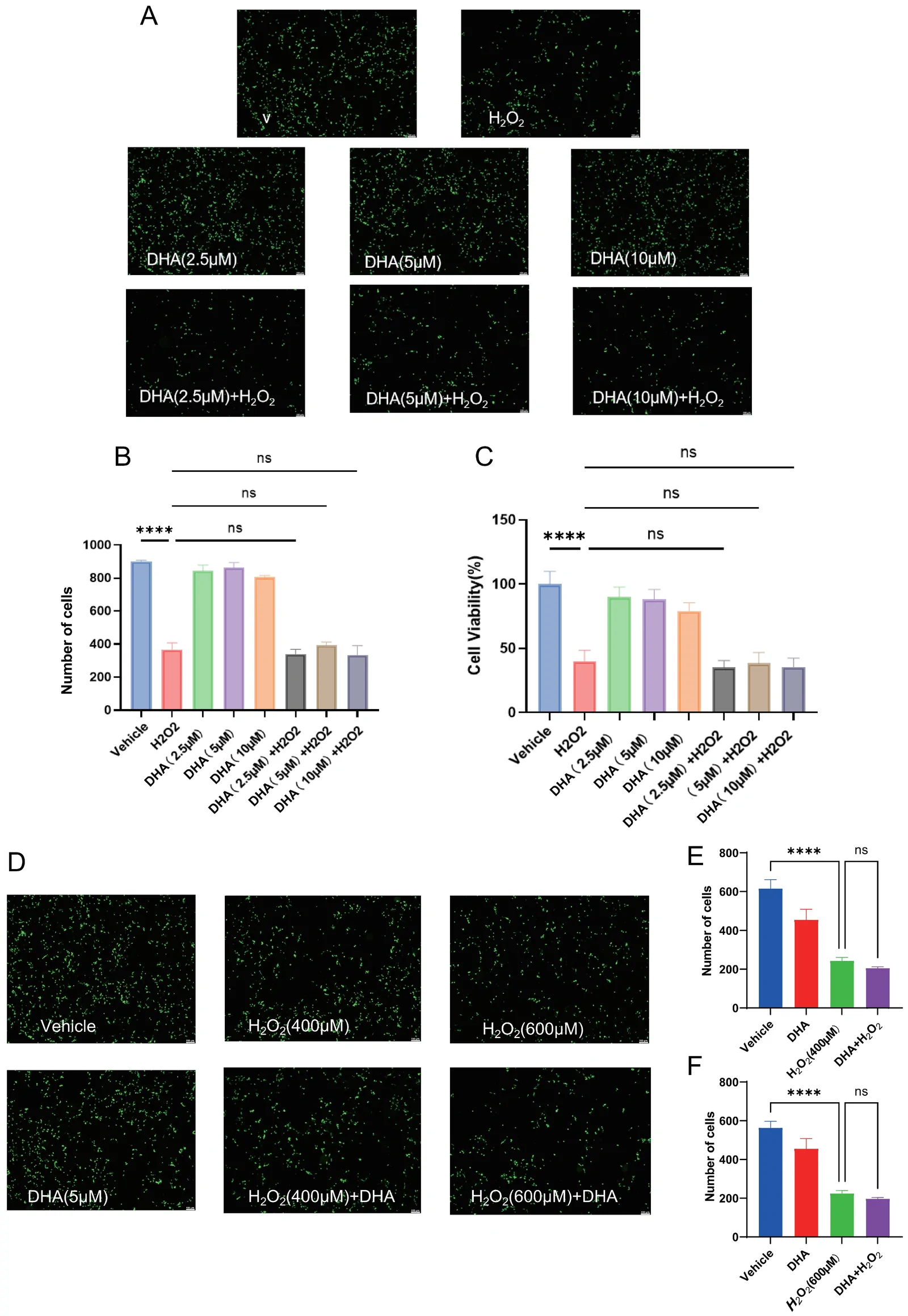
Effects of DHA on H_2_O_2_-induced oxidative injury in cIECs. (**A**) Representative Calcein-AM fluorescence images of cells in the Vehicle, 400 μM H_2_O_2_ alone, 2.5/5/10 μM DHA alone, and 2.5/5/10 μM DHA + 400 μM H_2_O_2_ groups. (**B**) Statistical quantification of viable cell counts across different DHA concentration gradients; (**C**) CCK-8-based cell viability detection for DHA concentration gradient. (**D**) Representative fluorescence images of cells in the Vehicle, 400 μM H_2_O_2_, 600 μM H_2_O_2_, 5 μM DHA alone, DHA + 400 μM H_2_O_2_, and DHA + 600 μM H_2_O_2_ groups. (**E**,**F**) Quantitative viable cell counts for 5 μM DHA intervention under 400 μM and 600 μM H_2_O_2_ challenge, respectively. Data are expressed as mean ± SD (*n* = 3 per group); ns, no significance, **** *p* < 0.0001. Scale bar = 100 μm.

To screen the optimal working concentration of EPA, three gradient concentrations (2.5, 5 and 10 μM) were set for pre-administration before H_2_O_2_ stimulation, followed by Calcein staining and CCK-8 cell viability detection (Figure 2A–C). Quantitative statistics demonstrated that 5 μM EPA achieved the maximal improvement in viability of damaged cIECs after oxidative insult, while 2.5 μM EPA showed partial protection and 10 μM EPA displayed no further increment in cytoprotection compared with the 5 μM group; accordingly, 5 μM EPA was selected for subsequent mechanistic assays.

Subsequent biochemical detection of oxidative stress-related indicators revealed dramatically elevated MDA content and significantly reduced enzymatic activities of total-SOD, CAT and GSH-Px in the H_2_O_2_-treated group relative to the Vehicle group (Figure 2D). After 5 μM EPA pretreatment, intracellular MDA accumulation in cIECs was efficiently restricted, accompanied by prominent recovery of SOD, CAT and GSH-Px activities (*p* < 0.05). Collectively, EPA effectively restored the impaired endogenous antioxidant system in oxidatively injured cIECs.

As shown in Figure 2A–C, no statistically significant difference in viable cell count or cell viability was observed between any DHA + H_2_O_2_ group and the H_2_O_2_ alone group (*p* > 0.05). We further tested the effect of 5 μM DHA under 400 μM and 600 μM H_2_O_2_ stimulation. Consistently, DHA pretreatment did not reverse H_2_O_2_-induced cell death at either concentration. No significant difference in viable cell number was detected between the DHA + H_2_O_2_ groups and the corresponding H_2_O_2_ alone groups (Figure 2D–F). These data collectively demonstrate that DHA exerts no detectable cytoprotective activity against H_2_O_2_-mediated oxidative injury in cIECs across all tested concentrations.

### 3.3. EPA Suppresses TNF-α and IFN-γ-Associated Inflammatory Signaling in cIECs

**Figure 3:**
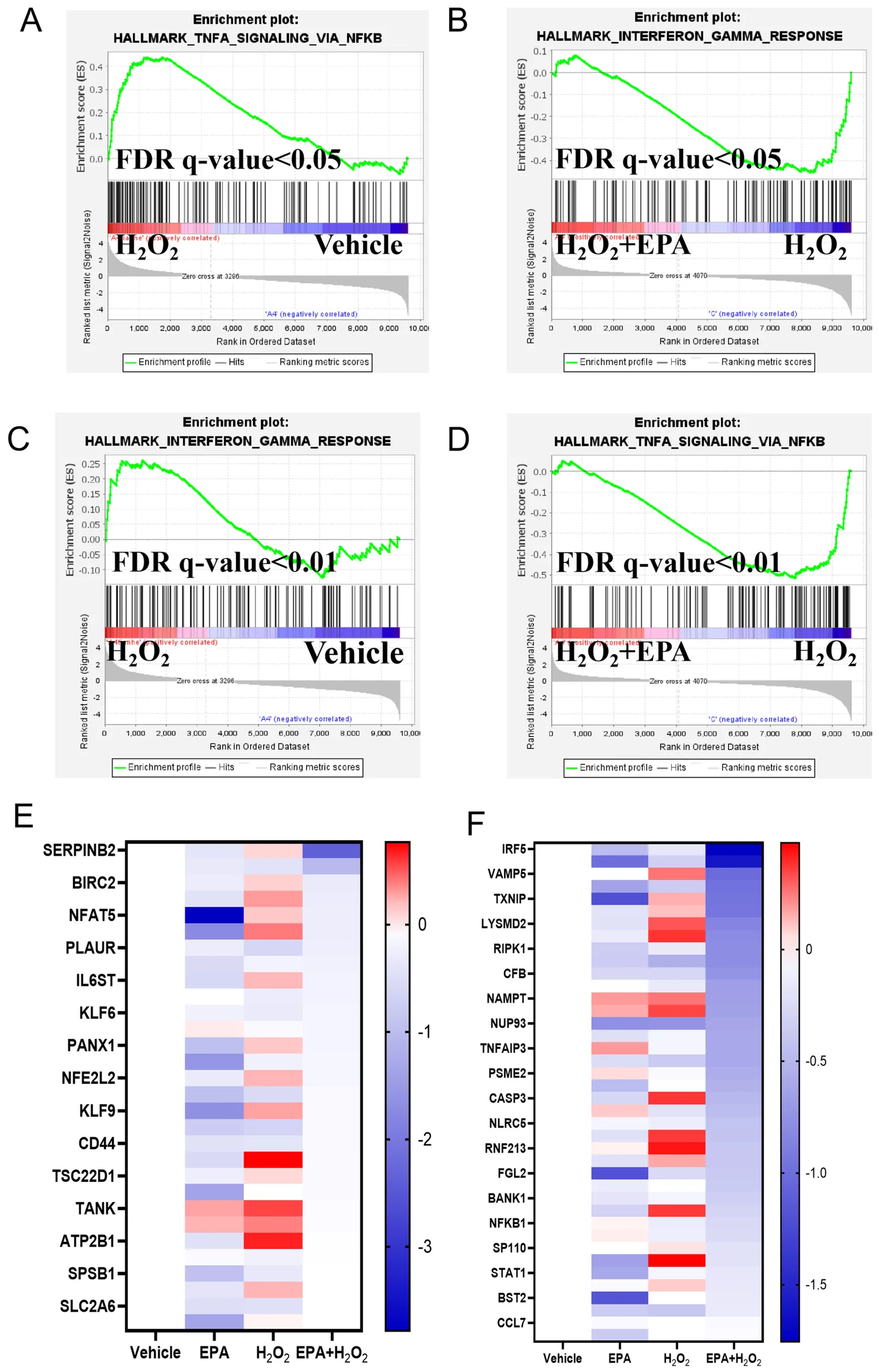
GSEA enrichment analysis of inflammatory pathways and heatmap of inflammation/antioxidant-related DEGs from cIECs. (**A**,**C**) GSEA enrichment plots of TNF-α/NF-κB and IFN-γ response signaling pathways for H_2_O_2_ vs. Vehicle comparison; (**B**,**D**) GSEA enrichment plots of TNF-α/NF-κB and IFN-γ response signaling pathways for EPA + H_2_O_2_ vs. H_2_O_2_ comparison; FDR q-value was labeled on each plot. (**E**,**F**) Heatmaps displaying normalized transcriptional expression of antioxidant-barrier-related genes (**E**) and innate immune/inflammation-related genes (**F**) among Vehicle, EPA, H_2_O_2_ and EPA + H_2_O_2_ four groups. Color gradient corresponds to normalized log_2_ fold change in gene expression.

Excessive ROS accumulation drives inflammatory activation; we thus investigated whether EPA mitigated H_2_O_2_-induced inflammation in cIECs. Transcriptome profiling combined with GSEA was conducted to decipher the transcriptional regulation of EPA on inflammatory pathways. GSEA results indicated that both TNF-α/NF-κB and IFN-γ response signaling pathways were significantly enriched and robustly upregulated in the H_2_O_2_ group versus Vehicle (FDR q < 0.05 or FDR q < 0.01; Figure 3A,C). Notably, EPA preconditioning effectively reversed the transcriptional overactivation of these two canonical pro-inflammatory pathways, as evidenced by markedly declined enrichment scores in the EPA + H_2_O_2_ group relative to the H_2_O_2_ group (FDR q < 0.05; Figure 3B,D).

Consistently, the heatmap of inflammation- and antioxidant barrier-associated differentially expressed genes (DEGs) further validated the above findings (Figure 3E,F). A large panel of NF-κB/IRF5-targeted pro-inflammatory genes (including *NFKB1*, *STAT1, IRF5, TNFAIP3, RIPK1* and CCL7) was drastically upregulated upon H_2_O_2_ treatment, whereas EPA supplementation reversed the aberrant upregulation of these inflammatory transcripts in cIECs. Meanwhile, NFE2L2 (encoding NRF2 protein, the master transcription factor governing cellular antioxidant response) and intestinal barrier-related genes (CD44, PLAUR) were abnormally induced after oxidative stress and normalized following EPA intervention.

### 3.4. EPA Alleviates H_2_O_2_-Induced Cellular Senescence in cIECs

**Figure 4:**
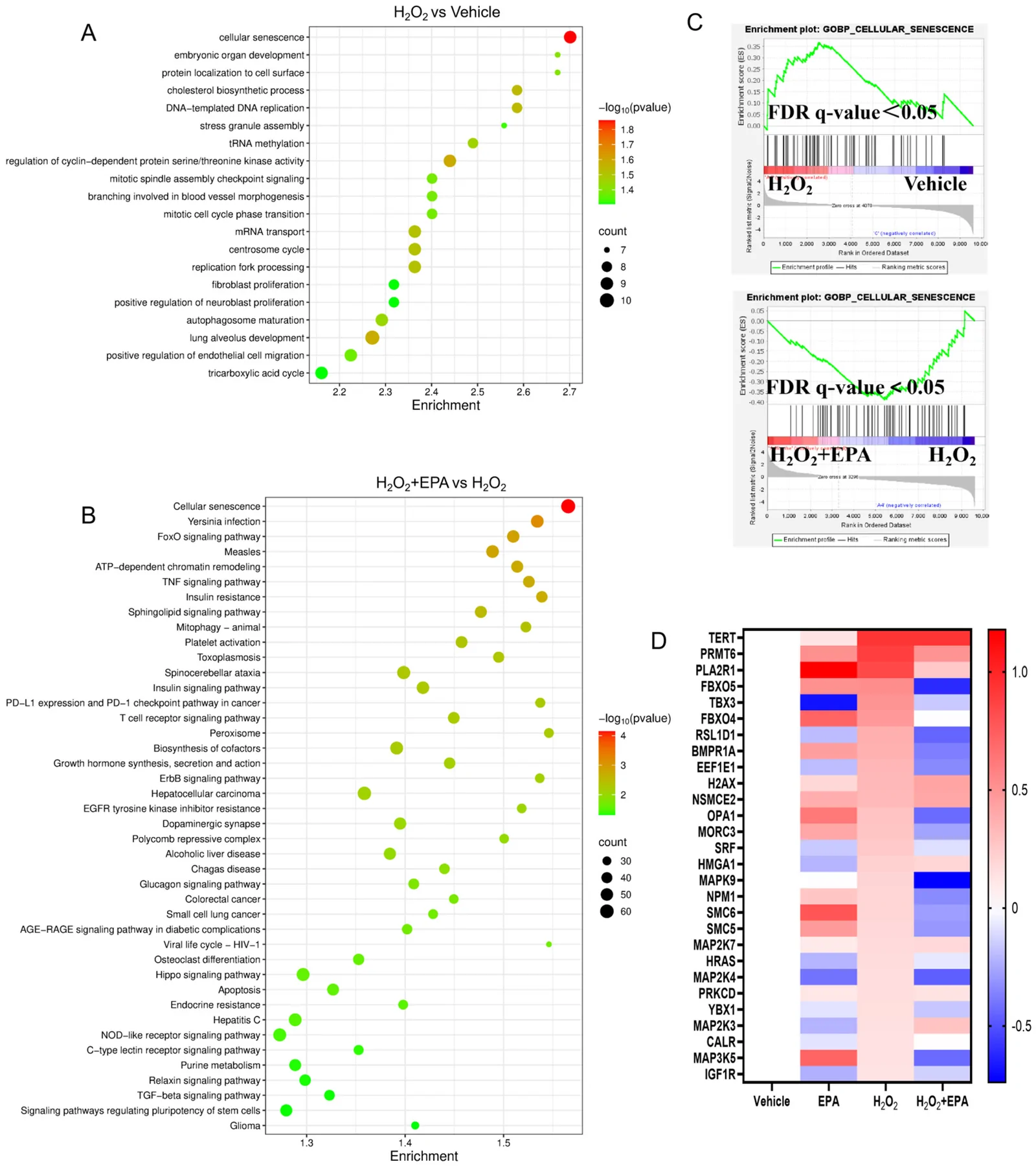
KEGG, senescence-related GSEA and DEGs heatmap of cIEC transcriptome. (**A**,**B**) KEGG enrichment plots of top altered pathways from two comparative sets: H_2_O_2_ vs. Vehicle (**A**) and EPA + H_2_O_2_ vs. H_2_O_2_ (**B**); *X*-axis = enrichment score, *Y*-axis = pathway name, bubble size = enriched DEG counts, color depth represents -log_10_(*p*-value). (**C**) GSEA enrichment curves of GO-based cellular senescence pathway for H_2_O_2_ vs. Vehicle (**upper**) and EPA + H_2_O_2_ vs. H_2_O_2_ (**lower**); FDR q < 0.05. (**D**) Heatmap showing relative expression of cellular senescence-associated DEGs across four experimental groups; the color scale indicates normalized gene expression level.

Further KEGG enrichment analysis was performed with two comparison cohorts to clarify pathway shifts (Figure 4A,B). KEGG results showed that cellular senescence represented one of the most significantly enriched biological pathways activated by H_2_O_2_ stimulation; conversely, cellular senescence was among the top enriched pathways significantly suppressed after EPA intervention in cIECs.

GSEA further verified the transcriptional alteration of cellular senescence signaling: the whole senescence-related gene set was remarkably upregulated in H_2_O_2_-challenged cIECs compared with Vehicle (FDR q < 0.05; Figure 4C upper), while EPA pretreatment markedly suppressed the overall expression of the senescence-related gene signature (FDR q < 0.05; Figure 4C lower). In line with enrichment results, the heatmap of senescence-associated DEGs illustrated that H_2_O_2_ induced robust upregulation of senescence-promoting transcripts, most of which were evidently downregulated upon EPA co-treatment in cIECs (Figure 4D).

### 3.5. EPA Alleviates Cellular Senescence and Restores Impaired Tight Junction Integrity in cIECs

**Figure 5:**
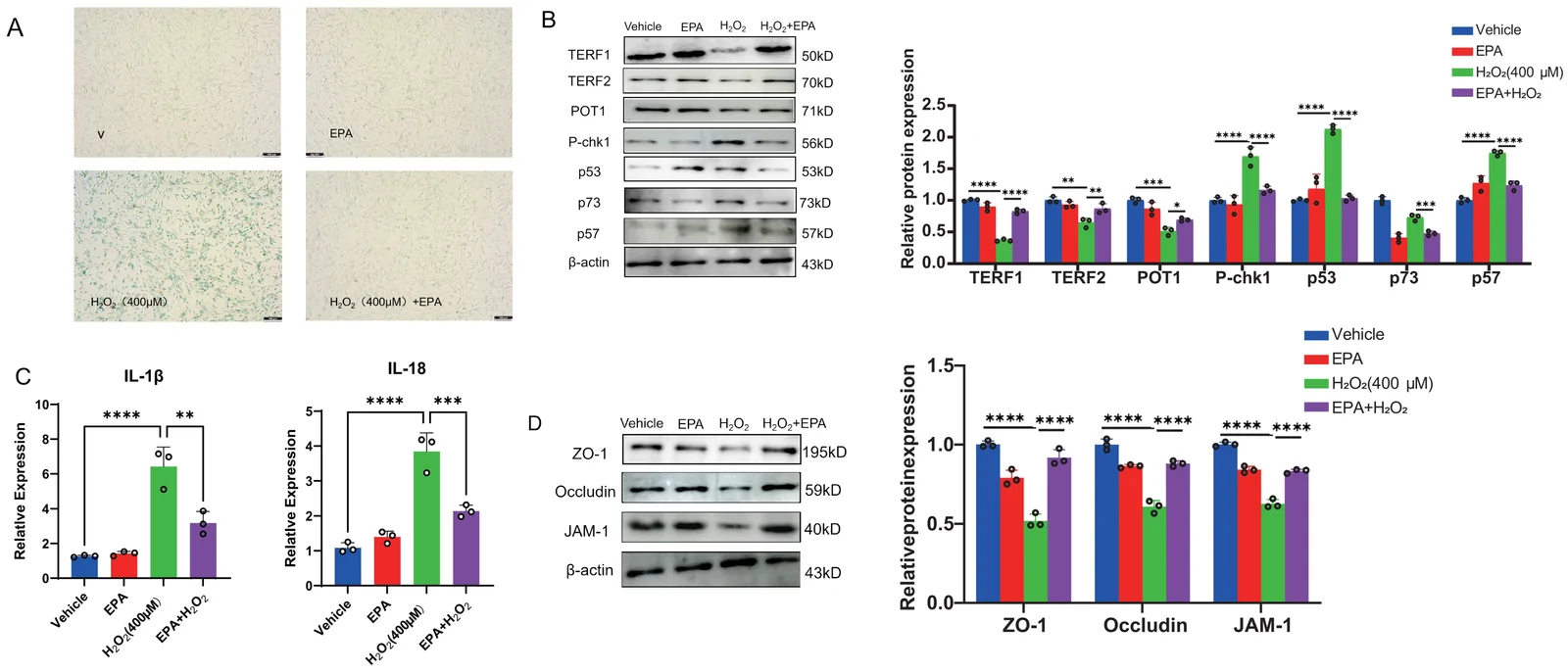
SA-β-gal senescence staining, Western blot and qPCR analysis of senescence and tight junction-associated molecules in cIECs. (**A**) Representative microscopic images of SA-β-gal staining for Vehicle, single EPA, H_2_O_2_ and EPA + H_2_O_2_ groups; blue staining marks senescent epithelial cells. (**B**) Western blot bands of telomere shelterin proteins (TERF1, TERF2, POT1) and senescence-regulatory proteins (P-chk1, p53, p57, p73), with β-actin as internal reference protein. (**C**) Relative mRNA expression levels of pro-senescent inflammatory cytokines *IL-1β* and *IL-18* quantified by qRT-PCR. (**D**) Western blot detection of core tight junction proteins ZO-1, Occludin and JAM1, β-actin served as loading control. Data are shown as mean ± SD (*n* = 3 per group); * *p* < 0.05, ** *p* < 0.01, *** *p* < 0.001, **** *p* < 0.0001.

Based on the above results, we further validated these protective effects via cytochemical staining and Western blotting. SA-β-galactosidase (SA-β-gal) staining was applied to visually assess the cellular senescence phenotype of cIECs (Figure 5A). Massive SA-β-gal-positive blue-stained senescent cells were observed in the H_2_O_2_ group, whereas the number of senescent cIECs was sharply reduced in the EPA + H_2_O_2_ combination group, and negligible positive signals were detected in the Vehicle and single EPA groups. Western blot assays for telomere-protective and senescence-regulatory proteins were then carried out (Figure 5B). H_2_O_2_ exposure markedly decreased the protein abundances of telomere shelterin complex components (TERF1, TERF2 and POT1) in cIECs, whereas it drastically elevated the expression of senescence-driving proteins (P-chk1, p53, p57 and p73). EPA pretreatment efficiently restored the expression of TERF1/TERF2/POT1 and downregulated the above senescence-related marker proteins. The complete original uncropped Western blot images can be found in Supplementary Figures S1–S8. Consistently, mRNA levels of senescence-associated pro-inflammatory cytokines IL-1β and IL-18 were significantly upregulated in the H_2_O_2_ group and markedly suppressed by EPA supplementation (*p* < 0.01; Figure 5C).

Inflammation and cellular senescence triggered by oxidative damage synergistically disrupt epithelial integrity and impair intestinal barrier function. Accordingly, we verified EPA’s protective effects and evaluated its efficacy to restore damaged tight junctions. The results of tight junction proteins demonstrated that H_2_O_2_ treatment caused obvious degradation of ZO-1, Occludin and JAM1 (Figure 5D), three core constituent proteins of epithelial tight junctions in cIECs. The complete original uncropped Western blot images can be found in Supplementary Figures S9–S12. Importantly, EPA preconditioning effectively recovered the protein expression of these tight junction proteins, contributing to the maintenance of intestinal epithelial barrier integrity.

## 4. Discussion

EPA is an ω-3 PUFA that cannot be endogenously synthesized in mammalian organisms and must be supplemented via daily diet, predominantly extracted from deep-sea fish oil and microalgal sources [15]. Its unique chemical structure consisting of 20 carbon chains and five cis-configured double bonds enables efficient insertion into cell membrane phospholipid bilayers, altering membrane fluidity and lipid microenvironment to modulate multiple membrane-binding signaling cascades, which is the structural basis for its diverse biological activities. Our preliminary concentration screening revealed a prominent phenotypic discrepancy between EPA and DHA under identical culture conditions: Under all equivalent gradient concentrations ranging from 2.5 to 10 μM in this experiment, 5 μM EPA significantly elevated the survival rate of H_2_O_2_-challenged canine ileal epithelial cells (cIECs), whereas DHA exhibited no obvious cytoprotective effects at any tested concentration, identifying the subtype-specific intestinal protective function of ω-3 PUFAs at the canine intestinal epithelial level for the first time. Consistent with previous nutritional investigations, EPA is inclined to participate in peripheral systemic immune modulation and inflammatory homeostasis across visceral tissues, whereas DHA preferentially accumulates in central nervous and retinal tissues to govern neurogenesis and visual development; such inherent tissue tropism partially accounts for their divergent efficacy against intestinal oxidative damage, yet the detailed transcriptional and post-translational regulatory gaps between the two fatty acids still require systematic exploration across diverse primary cell lines and animal models to clarify EPA’s dose-dependent physiological characteristics.

Combined RNA-sequencing and subsequent GSEA analysis elaborated the transcriptional regulatory mechanism by which EPA relieves H_2_O_2_-triggered cIEC damage. Oxidative stimulation robustly upregulated transcriptional activity of TNF-α and IFN-γ-mediated signaling pathways, two canonical upstream cascades initiating intestinal inflammatory responses, whereas EPA supplementation transcriptionally restrained the expression of abundant downstream pro-inflammatory target genes. These findings align with the well-established metabolic characteristic of EPA: it competitively displaces arachidonic acid (AA) from membrane phospholipids, limiting the generation of potent pro-inflammatory AA-derived eicosanoids and redirecting metabolic flux toward the synthesis of inflammation-resolving lipid mediators [16]. At the transcriptional level, EPA blocks the positive feedback loop between oxidative stress and inflammatory amplification, fundamentally cutting off the oxidative-inflammation vicious cycle and alleviating inflammatory erosion on intestinal epithelial structure. This indicates that EPA exerts anti-inflammatory protective effects at the transcriptional level by breaking the “oxidative stress–inflammation” vicious cycle.

Oxidative stress is a physiological imbalance that occurs when the antioxidant system cannot timely clear excessive ROS [17]. Excessive intracellular ROS accumulation disturbs endogenous antioxidant equilibrium, triggering epithelial apoptosis, tight junction fracture, and subsequent chronic intestinal inflammation in dogs [18,19]. MDA is an end product of lipid peroxidation and can serve as a biomarker for evaluating cellular oxidative damage [20]. And T-SOD, CAT, and GSH-Px cooperatively constitute the core enzymatic antioxidant defense system to eliminate redundant reactive oxygen metabolites [21,22,23]. Cumulative studies have validated that Nrf2/HO-1 signaling serves as the pivotal endogenous antioxidant axis to mitigate multi-organ oxidative impairment by activating downstream antioxidant enzyme transcription [24,25,26]. In our study, H_2_O_2_ intervention elevated MDA content and sharply suppressed the activities of three core antioxidant enzymes; conversely, EPA treatment effectively reversed these abnormal biochemical indices and lowered intracellular ROS accumulation, reconstructing the impaired antioxidant defense network of damaged cIECs and confirming EPA’s potent antioxidant capacity to maintain intestinal epithelial physiological homeostasis.

Oxidative stress-induced telomere attrition and DNA double-strand breakage are core inducers of irreversible cellular senescence, which is characterized by permanent cell cycle arrest, positive SA-β-gal staining and robust senescence-associated secretory phenotype (SASP) secretion; secreted pro-inflammatory cytokines further induce paracrine senescence of adjacent epithelial cells and deteriorate the intestinal microenvironment [27,28,29]. The Shelterin complex composed of TERF1, TERF2 and POT1 maintains telomere structural stability [30]; stress-induced complex dissociation initiates DNA damage response, elevates phosphorylated Chk1 and sequentially activates p53/p57/p73 checkpoint cascades to arrest cell cycle progression [31]. Our results indicated H_2_O_2_ treatment triggered an obvious senescent phenotype in cIECs accompanied by downregulated Shelterin proteins and upregulated senescence-related regulatory proteins, whereas EPA restored telomere-protective protein expression and suppressed p-Chk1, p53, p57, p73 as well as SASP-related IL-1β and IL-18 expression. This evidence confirms EPA delays intestinal cell senescence via stabilizing telomere architecture and intercepting DNA damage-initiated cell cycle arrest. Furthermore, impaired telomere function could compromise intestinal barrier integrity [29]. Intestinal physical barrier integrity depends on intact epithelial monolayer and intercellular tight junctions (TJs), with ZO-1, Occludin and JAM-1 functioning as core structural proteins to block paracellular pathogen translocation and regulate nutrient permeability [32]. H_2_O_2_-induced oxidative injury significantly downregulated these tight junction proteins, while EPA effectively recovered their expression to preserve barrier integrity, providing direct molecular evidence supporting EPA’s intestinal protective property.

It should be objectively acknowledged that gene knockout or specific inhibitor intervention assays were not performed in the present study. Given that EPA exhibits multi-target regulatory effects and its underlying mechanisms are complex, we will systematically dissect the causal regulatory mechanisms through which EPA sustains canine intestinal health in subsequent research by combining in vivo canine feeding models with targeted gene silencing techniques.

## 5. Conclusions

In conclusion, the present in vitro study using canine ileal epithelial cells (cIECs) confirmed distinct functional differences between two typical ω-3 polyunsaturated fatty acids: Under all tested gradient concentrations from 2.5 to 10 μM in this experiment, equivalent concentration of DHA failed to alleviate H_2_O_2_-provoked cell damage, whereas EPA exerted prominent multi-target protective activities against oxidative injury. Mechanistically, EPA elevates cellular antioxidant enzyme activities to eliminate redundant ROS and reverse redox homeostasis disorder, thereby improving the survival rate of oxidatively damaged cIECs. At the transcriptional level, EPA suppresses aberrant activation of TNF-α and IFN-γ pro-inflammatory signaling cascades to prevent the vicious loop between oxidative stress and inflammation. Meanwhile, EPA stabilizes telomere shelterin complex (TERF1, TERF2, POT1) expression, inhibits the activation of DNA damage marker p-Chk1 and downstream senescence-related proteins p53, p57 and p73, and further reduces the secretion of senescence-associated pro-inflammatory cytokines, ultimately restraining oxidative stress-triggered cellular senescence. In addition, EPA recovers the expression of tight junction proteins ZO-1, Occludin and JAM-1 to reconstruct the impaired intestinal epithelial physical barrier. Collectively, these data demonstrate that EPA is a promising natural functional feed additive to mitigate intestinal oxidative damage, retard epithelial senescence and sustain intestinal barrier function in companion dogs. Further in vivo feeding trials are required to optimize practical supplemental dosage, investigate synergistic interactions between EPA and other dietary nutrients, and translate these cellular findings into clinical application for canine intestinal health regulation.

## Data Availability

The raw data supporting the conclusions of this article will be made available by the authors on request.

## Supplementary Materials

The following supporting information can be downloaded at: https://www.mdpi.com/article/10.3390/vetsci13080765/s1, Figure S1. Original uncropped western blot image of β-actin. Four experimental groups were loaded in order: Vehicle, EPA, H_2_O_2_, H_2_O_2_ + EPA. The expected molecular weight of β-actin is 43 kDa. Primary antibody: β-actin (1:1000, mouse); Secondary antibody: anti-mouse IgG (1:2000). Uniform brightness and contrast adjustment was applied to the whole blot for visualization. All blots were repeated independently ≥3 times. Figure S2. Original uncropped western blot image of TERF1. Four experimental groups were loaded in order: Vehicle, EPA, H_2_O_2_, H_2_O_2_ + EPA. The expected molecular weight of TERF1 is 50 kDa. Primary antibody: TERF1 (1:1000, rabbit); Secondary antibody: anti-rabbit IgG (1:2000). Uniform brightness and contrast adjustment was applied to the whole blot for visualization. All blots were repeated independently ≥3 times. Figure S3. Original uncropped western blot image of TERF2. Four experimental groups were loaded in order: Vehicle, EPA, H_2_O_2_, H_2_O_2_ + EPA. The expected molecular weight of TERF2 is 70 kDa. Primary antibody: TERF2 (1:1000, rabbit); Secondary antibody: anti-rabbit IgG (1:2000). Uniform brightness and contrast adjustment was applied to the whole blot for visualization. All blots were repeated independently ≥3 times. Figure S4. Original uncropped western blot image of POT1. Four experimental groups were loaded in order: Vehicle, EPA, H_2_O_2_, H_2_O_2_ + EPA. The expected molecular weight of POT1 is 71 kDa. Primary antibody: POT1 (1:1000, rabbit); Secondary antibody: anti-rabbit IgG (1:2000). Uniform brightness and contrast adjustment was applied to the whole blot for visualization. All blots were repeated independently ≥3 times. Figure S5. Original uncropped western blot image of phosphorylated Chk1 (P-chk1). Four experimental groups were loaded in order: Vehicle, EPA, H_2_O_2_, H_2_O_2_ + EPA. The expected molecular weight of P-chk1 is 56 kDa. Primary antibody: P-chk1 (1:1000, rabbit); Secondary antibody: anti-rabbit IgG (1:2000). Uniform brightness and contrast adjustment was applied to the whole blot for visualization. All blots were repeated independently ≥3 times. Figure S6. Original uncropped western blot image of p53. Four experimental groups were loaded in order: Vehicle, EPA, H_2_O_2_, H_2_O_2_ + EPA. The expected molecular weight of p53 is 53 kDa. Primary antibody: p53 (1:1000, rabbit); Secondary antibody: anti-rabbit IgG (1:2000). Uniform brightness and contrast adjustment was applied to the whole blot for visualization. All blots were repeated independently ≥3 times. Figure S7. Original uncropped western blot image of p73. Four experimental groups were loaded in order: Vehicle, EPA, H_2_O_2_, H_2_O_2_ + EPA. The expected molecular weight of p73 is 73 kDa. Primary antibody: p73 (1:1000, rabbit); Secondary antibody: anti-rabbit IgG (1:2000). Uniform brightness and contrast adjustment was applied to the whole blot for visualization. All blots were repeated independently ≥3 times. Figure S8. Original uncropped western blot image of p57. Four experimental groups were loaded in order: Vehicle, EPA, H_2_O_2_, H_2_O_2_ + EPA. The expected molecular weight of p57 is 57 kDa. Primary antibody: p57 (1:1000, rabbit); Secondary antibody: anti-rabbit IgG (1:2000). Uniform brightness and contrast adjustment was applied to the whole blot for visualization. All blots were repeated independently ≥3 times. Figure S9. Original uncropped western blot image of ZO-1. Four experimental groups were loaded in order: Vehicle, EPA, H_2_O_2_, H_2_O_2_ + EPA. The expected molecular weight of ZO-1 is 195 kDa. Primary antibody: ZO-1 (1:1000, rabbit); Secondary antibody: anti-rabbit IgG (1:2000). Uniform brightness and contrast adjustment was applied to the whole blot for visualization. All blots were repeated independently ≥3 times. Figure S10. Original uncropped western blot image of Occludin. Four experimental groups were loaded in order: Vehicle, EPA, H_2_O_2_, H_2_O_2_ + EPA. The expected molecular weight of Occludin is 59 kDa. Primary antibody: Occludin (1:1000, rabbit); Secondary antibody: anti-rabbit IgG (1:2000). Uniform brightness and contrast adjustment was applied to the whole blot for visualization. All blots were repeated independently ≥3 times. Figure S11. Original uncropped western blot image of JAM-1. Four experimental groups were loaded in order: Vehicle, EPA, H_2_O_2_, H_2_O_2_ + EPA. The expected molecular weight of JAM-1 is 40 kDa. Primary antibody: JAM-1 (1:1000, rabbit); Secondary antibody: anti-rabbit IgG (1:2000). Uniform brightness and contrast adjustment was applied to the whole blot for visualization. All blots were repeated independently ≥3 times. Figure S12. Original uncropped western blot image of β-actin. Four experimental groups were loaded in order: Vehicle, EPA, H_2_O_2_, H_2_O_2_ + EPA. The expected molecular weight of β-actin is 43 kDa. Primary antibody: β-actin (1:1000, mouse); Secondary antibody: anti-mouse IgG (1:2000). Uniform brightness and contrast adjustment was applied to the whole blot for visualization. All blots were repeated independently ≥3 times..
