# Supplementary material for: Eicosapentaenoic Acid (EPA) Alleviates Oxidative Stress and Inflammatory Responses in Canine Ileal Epithelial Cells
Source: Vet Sci. 2026 Jul 31;13(8):765. doi: 10.3390/vetsci13080765 (PMC13517765; doi:10.3390/vetsci13080765)
Supplement: Supplementary file 1 [file vetsci-13-00765-s001.zip › vetsci-4390250-supplementary.pdf]

β-actin

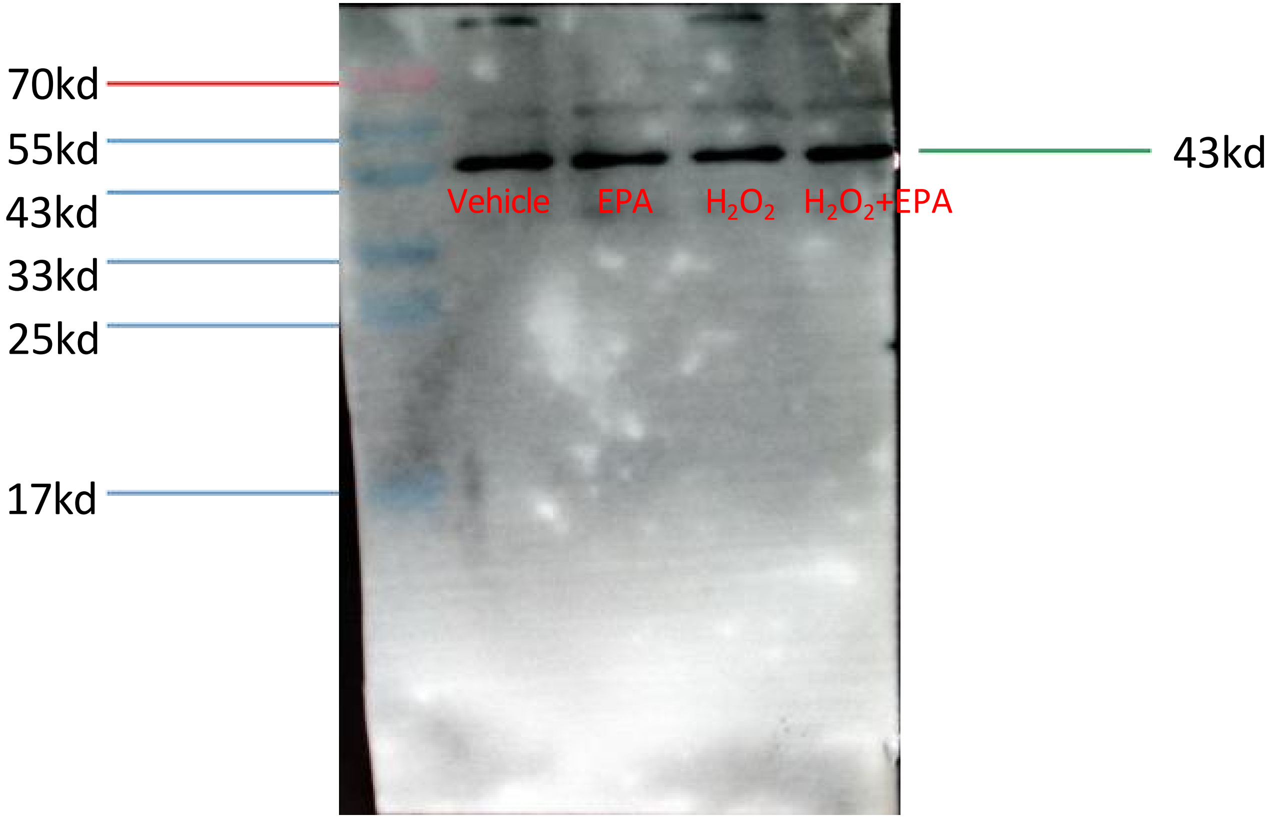

Figure S1. Original uncropped western blot image of β-actin. Four experimental groups were loaded in order: Vehicle, EPA, H_2O_2, H_2O_2+EPA. The expected molecular weight of β-actin is 43 kDa. Primary antibody: β-actin (1:1000, mouse); Secondary antibody: anti-mouse IgG (1:2000). Uniform brightness and contrast adjustment was applied to the whole blot for visualization. All blots were repeated independently ≥3 times.

TERF1

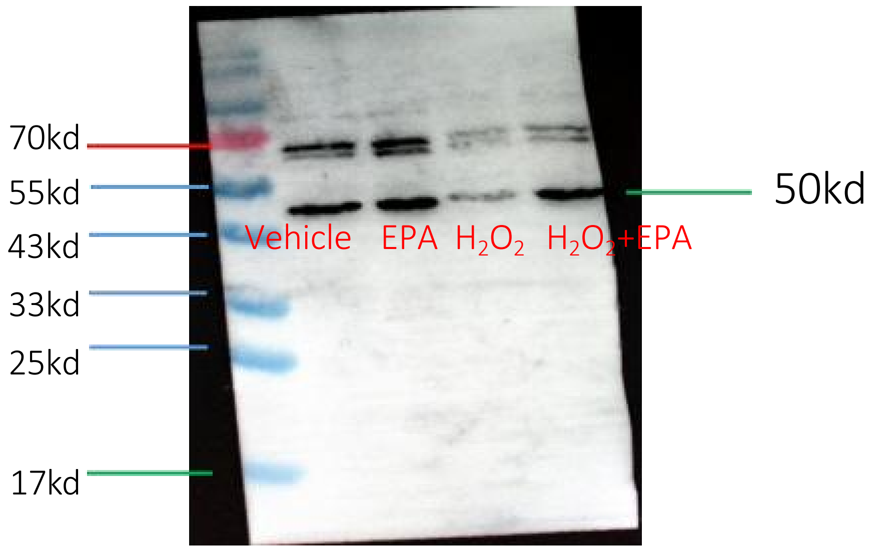

Figure S2. Original uncropped western blot image of TERF1. Four experimental groups were loaded in order: Vehicle, EPA, H_2O_2, H_2O_2+EPA. The expected molecular weight of TERF1 is 50 kDa. Primary antibody: TERF1 (1:1000, rabbit); Secondary antibody: anti-rabbit IgG (1:2000). Uniform brightness and contrast adjustment was applied to the whole blot for visualization. All blots were repeated independently ≥3 times.

TERF2

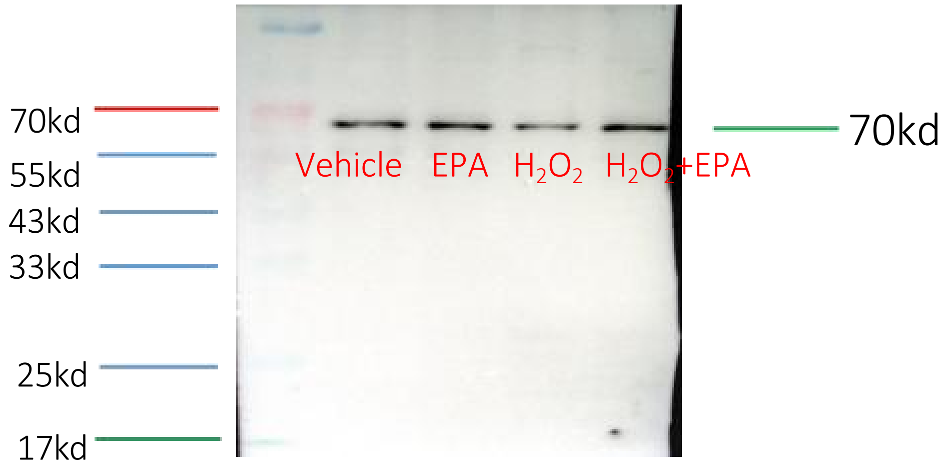

Figure S3. Original uncropped western blot image of TERF2. Four experimental groups were loaded in order: Vehicle, EPA, H_2O_2, H_2O_2+EPA. The expected molecular weight of TERF2 is 70 kDa. Primary antibody: TERF2 (1:1000, rabbit); Secondary antibody: anti-rabbit IgG (1:2000). Uniform brightness and contrast adjustment was applied to the whole blot for visualization. All blots were repeated independently ≥3 times.

POT1

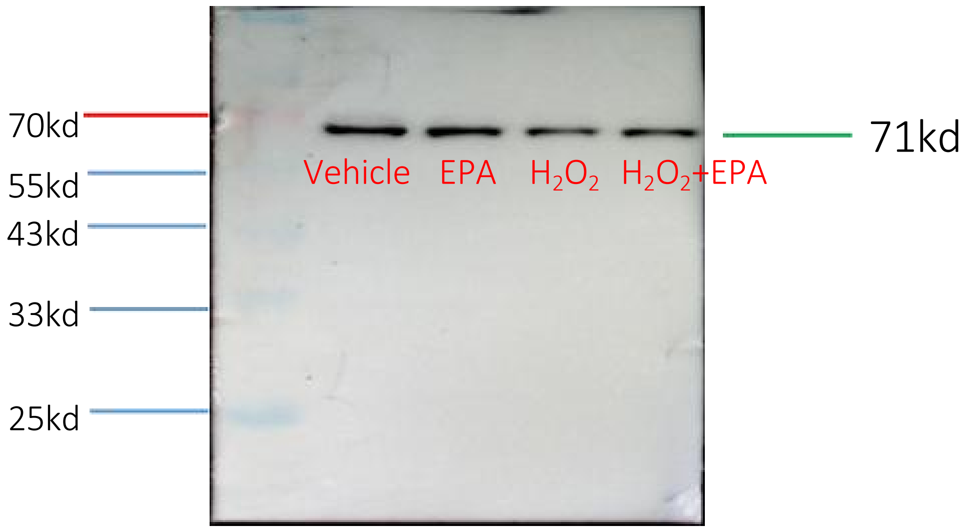

Figure S4. Original uncropped western blot image of POT1. Four experimental groups were loaded in order: Vehicle, EPA, H_2O_2, H_2O_2+EPA. The expected molecular weight of POT1 is 71 kDa. Primary antibody: POT1 (1:1000, rabbit); Secondary antibody: anti-rabbit IgG (1:2000). Uniform brightness and contrast adjustment was applied to the whole blot for visualization. All blots were repeated independently ≥3 times.

P-chk1

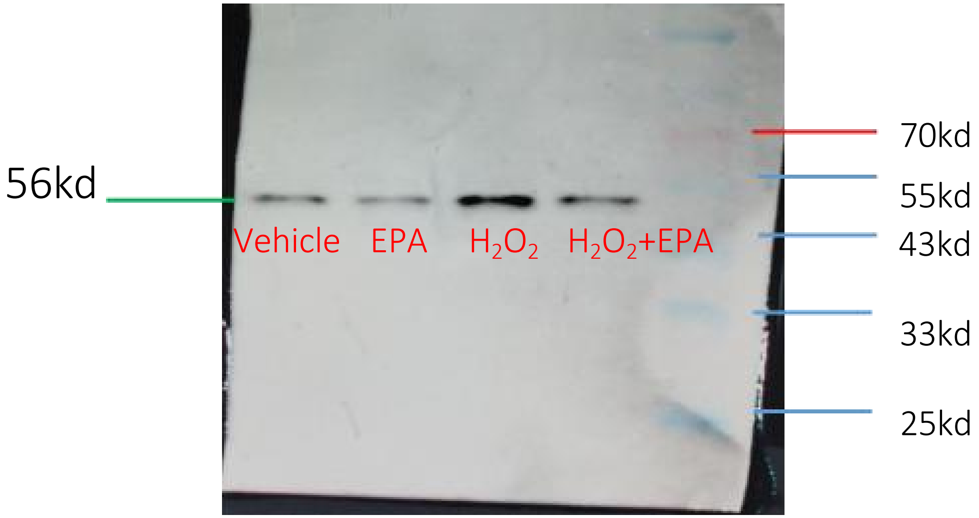

Figure S5. Original uncropped western blot image of phosphorylated Chk1 (P-chk1). Four experimental groups were loaded in order: Vehicle, EPA, H_2O_2, H_2O_2+EPA. The expected molecular weight of P-chk1 is 56 kDa. Primary antibody: P-chk1 (1:1000, rabbit); Secondary antibody: anti-rabbit IgG (1:2000). Uniform brightness and contrast adjustment was applied to the whole blot for visualization. All blots were repeated independently ≥3 times.

p53

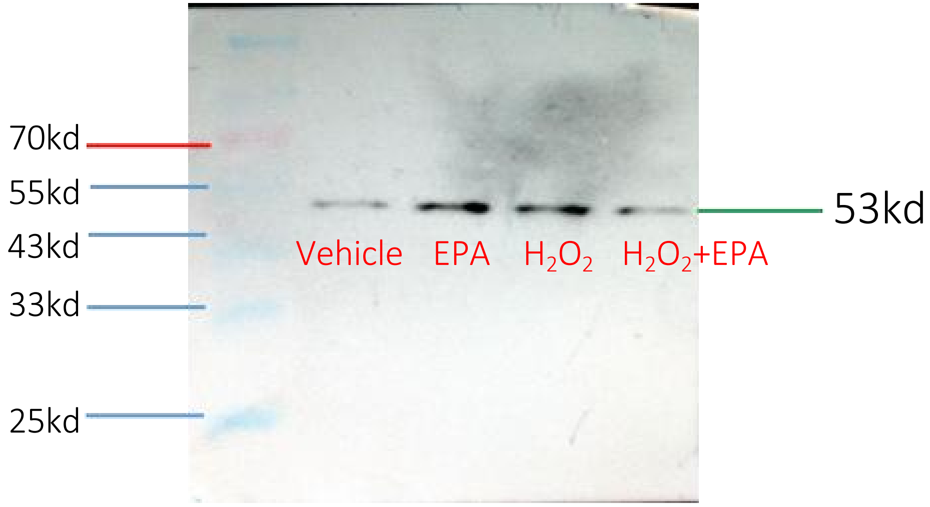

Figure S6. Original uncropped western blot image of p53. Four experimental groups were loaded in order: Vehicle, EPA, H_2O_2, H_2O_2+EPA. The expected molecular weight of p53 is 53 kDa. Primary antibody: p53 (1:1000, rabbit); Secondary antibody: anti-rabbit IgG (1:2000). Uniform brightness and contrast adjustment was applied to the whole blot for visualization. All blots were repeated independently ≥3 times.

p73

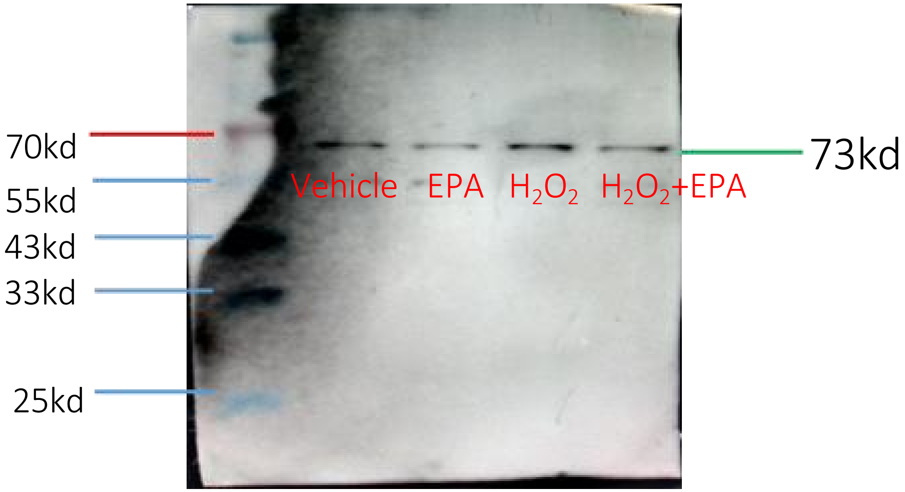

Figure S7. Original uncropped western blot image of p73. Four experimental groups were loaded in order: Vehicle, EPA, H_2O_2, H_2O_2+EPA. The expected molecular weight of p73 is 73 kDa. Primary antibody: p73 (1:1000, rabbit); Secondary antibody: anti-rabbit IgG (1:2000). Uniform brightness and contrast adjustment was applied to the whole blot for visualization. All blots were repeated independently ≥3 times.

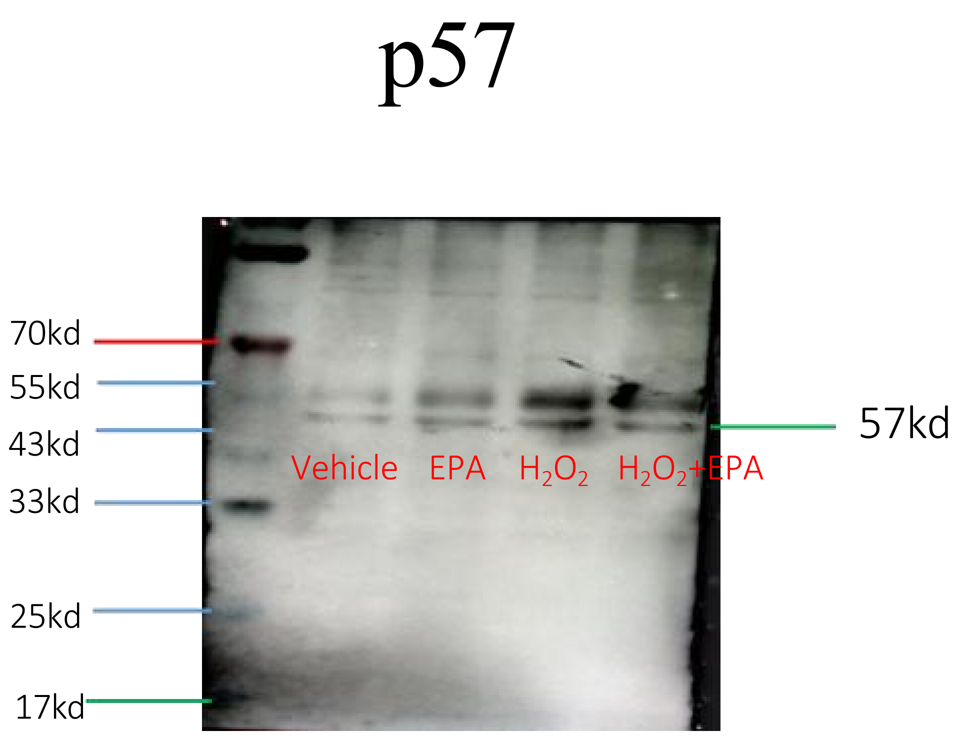

Figure S8. Original uncropped western blot image of p57. Four experimental groups were loaded in order: Vehicle, EPA, H_2O_2, H_2O_2+EPA. The expected molecular weight of p57 is 57 kDa. Primary antibody: p57 (1:1000, rabbit); Secondary antibody: anti-rabbit IgG (1:2000). Uniform brightness and contrast adjustment was applied to the whole blot for visualization. All blots were repeated independently ≥3 times.

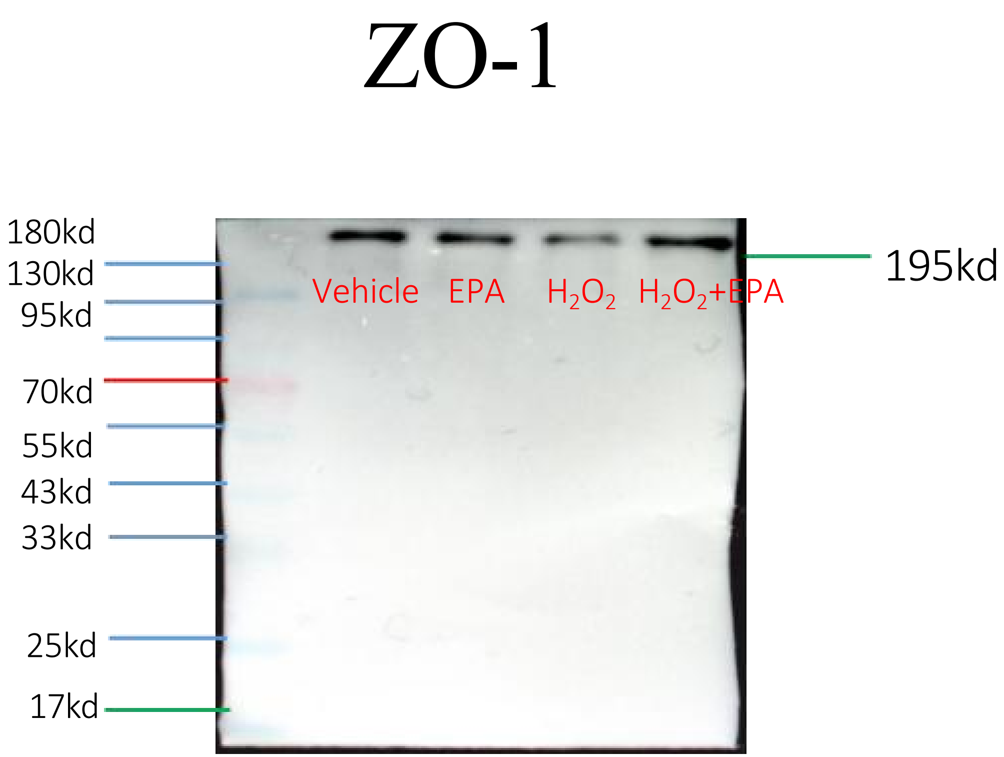

Figure S9. Original uncropped western blot image of ZO-1. Four experimental groups were loaded in order: Vehicle, EPA, H_2O_2, H_2O_2+EPA. The expected molecular weight of ZO-1 is 195 kDa. Primary antibody: ZO-1 (1:1000, rabbit); Secondary antibody: anti-rabbit IgG (1:2000). Uniform brightness and contrast adjustment was applied to the whole blot for visualization. All blots were repeated independently ≥3 times.

Occludin

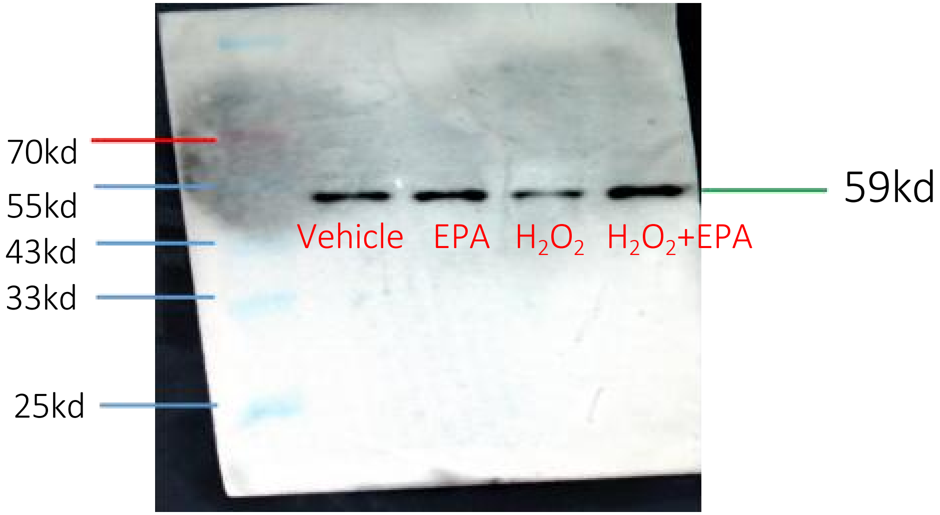

Figure S10. Original uncropped western blot image of Occludin. Four experimental groups were loaded in order: Vehicle, EPA, H_2O_2, H_2O_2+EPA. The expected molecular weight of Occludin is 59 kDa. Primary antibody: Occludin (1:1000, rabbit); Secondary antibody: anti-rabbit IgG (1:2000). Uniform brightness and contrast adjustment was applied to the whole blot for visualization. All blots were repeated independently ≥3 times.

JAM-1

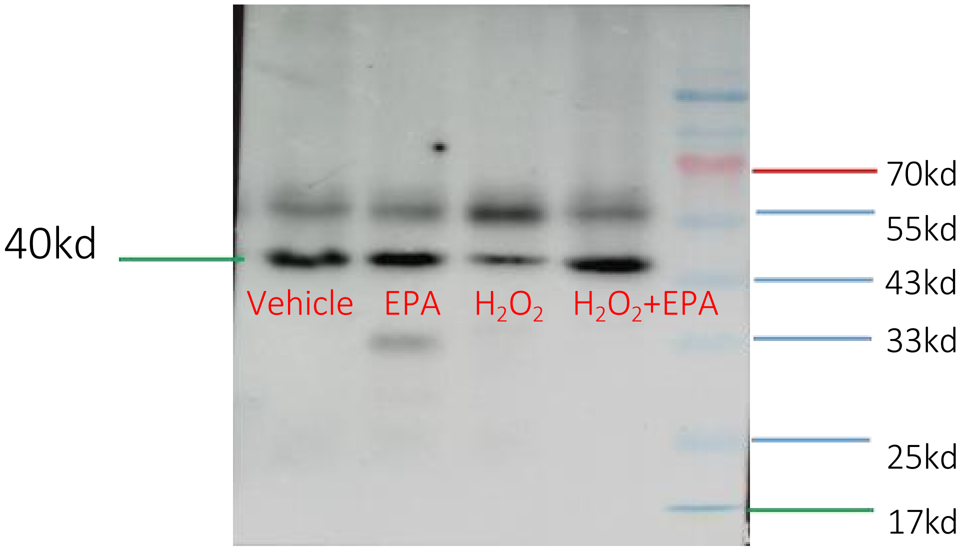

Figure S11. Original uncropped western blot image of JAM-1. Four experimental groups were loaded in order: Vehicle, EPA, H_2O_2, H_2O_2+EPA. The expected molecular weight of JAM-1 is 40 kDa. Primary antibody: JAM-1 (1:1000, rabbit); Secondary antibody: anti-rabbit IgG (1:2000). Uniform brightness and contrast adjustment was applied to the whole blot for visualization. All blots were repeated independently ≥3 times.

β-actin

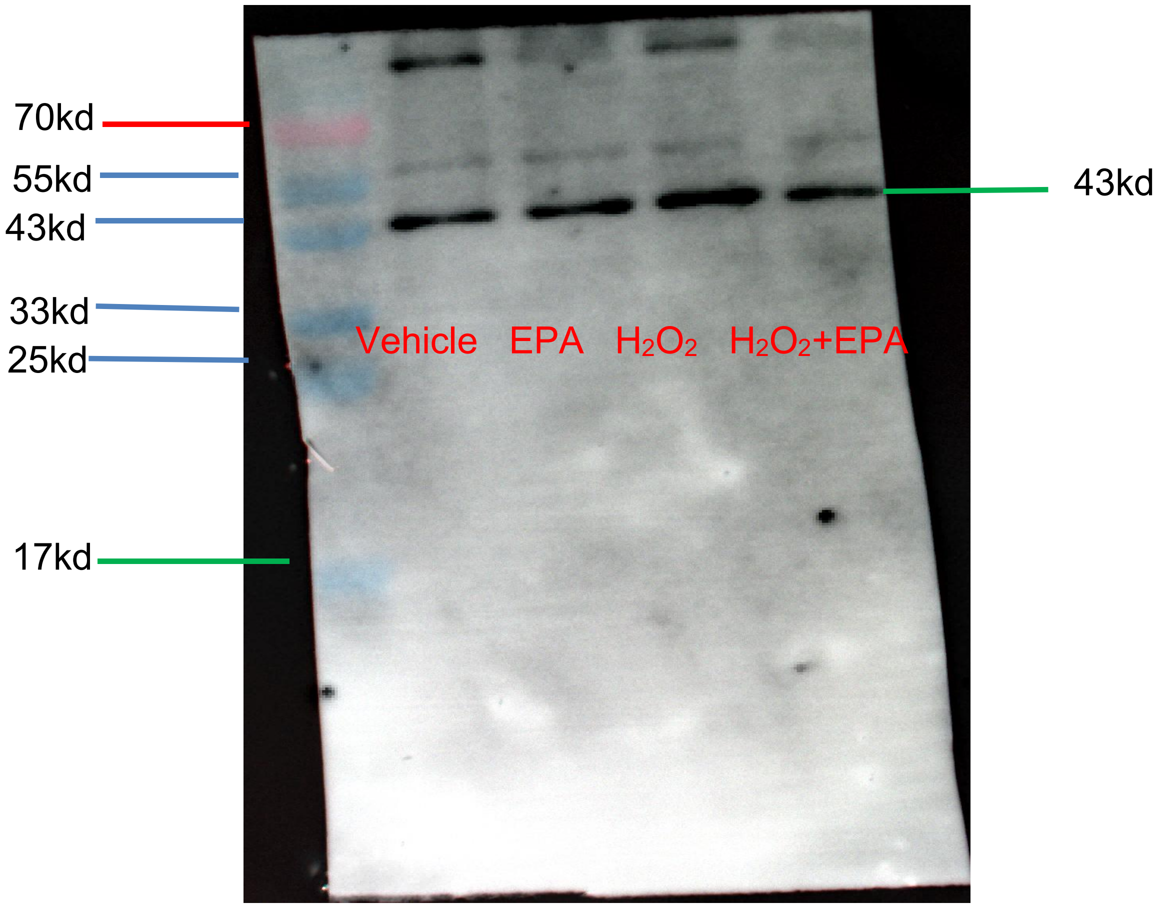

Figure S12. Original uncropped western blot image of β-actin. Four experimental groups were loaded in order: Vehicle, EPA, H_2O_2, H_2O_2+EPA. The expected molecular weight of β-actin is 43 kDa. Primary antibody: β-actin (1:1000, mouse); Secondary antibody: anti-mouse IgG (1:2000). Uniform brightness and contrast adjustment was applied to the whole blot for visualization. All blots were repeated independently ≥3 times.
